# Supplementary material for: Collaborative intelligence in AI: Evaluating the performance of a council of AIs on the USMLE
Source: PLOS Digit Health. 2025 Oct 9;4(10):e0000787. doi: 10.1371/journal.pdig.0000787 (PMC12510544; doi:10.1371/journal.pdig.0000787)
Supplement: S2 File — (ZIP) [file pdig.0000787.s002.zip › S2 Data Raw data files containing unprocessed question inputs and LLM outputs including transcripts of deliberations/STEP3_Question-112-1_Type-MC-NJ.docx]

**Facilitator** **[Round 1]:** A 24-year-old man comes to the emergency department because of right lower quadrant abdominal pain and nausea that began suddenly 6 hours ago. He rates the pain as a 6 on a 10-point scale and asks for pain medication. Medical history is unremarkable. He takes only a multivitamin. Vital signs on arrival are temperature 38.7°C (101.6°F), pulse 105/min, respirations 16/min, and blood pressure 110/85 mm Hg. Physical examination discloses exquisite tenderness in the right lower quadrant of the abdomen. The remainder of the examination discloses no abnormalities. The surgical consultant cannot evaluate the patient for 2 hours. Which of the following is the most appropriate next step? (A) Administer acetaminophen (B) Administer gabapentin (C) Administer lorazepam (D) Administer morphine (E) Explain to the patient that analgesics would interfere with the surgical examination

**AI_C:** RESTATING THE QUESTION: We're discussing the case of a 24-year-old man who presented to the emergency department with right lower quadrant abdominal pain, fever, increased heart rate, and nausea, and the surgical consultant cannot evaluate the patient for a few hours. The question is asking what is the most appropriate next step to take. PLANNING: The assumptions here are that the individual has been suffering from an unspecified abdominal condition for 6 hours that could possibly be appendicitis, and that managing his pain is the immediate priority. A significant assumption is that analgesics might obstruct subsequent examinations. This can be questioned and evaluated through our knowledge of emergency pain management and abdominal conditions. CLINICAL REASONING: It has been a common belief that administering analgesics to a patient complaining of abdominal pain might mask the pain, thereby obscuring the diagnosis. However, recent studies show that the fear of masking the pain and delaying the diagnosis of appendicitis with analgesics is unfounded. Pain relief won’t reduce the tenderness on palpation, nor will it confuse the clinical picture or delay the diagnosis. Patients with pain should receive pain treatment, and this patient is clearly in pain. CONCLUDING: In this case, the patient has to wait for two hours before any surgical intervention can take place. It is essential during this time to manage his pain, both for his comfort and to prevent further stress and anxiety from the severe pain he is experiencing. RESPONSE: The most appropriate next step would be to "Administer morphine" (Option D). This provides immediate and effective relief for the patient's pain. Analgesics would not interfere with the surgical examination or jeopardize the patient's health in this scenario.

**AI_B:** RESTATING THE QUESTION: This clinical scenario is presenting a 24-year-old man who is experiencing sudden right lower quadrant abdominal pain and nausea, which he rates a 6 out of 10. The examination reveals tenderness in the right lower quadrant of his abdomen and his vitals indicate significant inflammation (elevated temperature indicating possible fever). A surgical consultation will occur in 2 hours. We're asked to determine an immediate step to help manage the patient’s condition. PLANNING: This clinical presentation suggests acute appendicitis, given the sudden onset of right lower quadrant pain and accompanying symptoms such as fever and nausea. Several assumptions can be made from these descriptions: (1) the pain may be severe hence the urge to request for analgesia, (2) the inflammation may be progressing, and (3) the time taken prior to surgical procedure is crucial for pain management. Alternate assumptions could include various pain management strategies, such as using analgesic medications, or possibly not administering any if it may interfere with surgical examination. CLINICAL REASONING: It's essential to consider the mechanism of action and the potential global effects of the medications. Acetaminophen and morphine are analgesics, lorazepam is an anxiolytic and has sedative effects, and gabapentin has antiepileptic and neuropathic pain benefits. Given the mechanism of action for these medications and understanding that an analgesic may be warranted due to the severity of the pain, the clinical priorities addressed concern patient comfort and maintaining a viable clinical picture of the patient's condition for the surgical consult. CONCLUDING: It seems like the most appropriate next treatment step would not compromise the surgical consult's ability to diagnose and evaluate the patient while providing some form of relief for the patient's pain. Although the use of analgesics in acute abdominal pain was traditionally avoided to maintain the clinical picture, recent evidence suggests they don't obscure the diagnosis and can improve patient comfort. RESPONSE: (D) Administer morphine: This medication will provide the necessary pain relief for the patient while waiting for the surgical consultation. It falls within acceptable practice according to recent evidence and will improve patient comfort without significantly disrupting the ability of the surgical consultant to evaluate the patient's condition.

**AI_D:** RESTATING THE QUESTION: To rephrase the provided scenario, a young man has appeared in the emergency department, presenting with sudden, intense right lower quadrant abdominal pain, nausea, and elevated temperature. His medical history doesn't reveal any significant facts. His vitals indicate fever and a slightly increased heart rate. The abdominal examination reveals acute tenderness localized in the right lower quadrant, with no other anomalies noted. There will be a delay before a surgical consultant can evaluate him. We need to determine the best course of action to take next, with options including different types of pain relief or informing the patient that analgesics would potentially interfere with his upcoming surgical examination. PLANNING: Current assumptions include acute appendicitis as a potential diagnosis due to the common symptom of right lower quadrant abdominal pain and nausea. Given the symptoms, the clinical priority would be to manage the patient's pain while confirming this diagnosis. Pain medication may indeed interfere with the surgical evaluation; however, allowing the patient to continue experiencing severe pain would infringe upon the ethical principle of beneficence, which prioritizes patient welfare. Several medications are options: acetaminophen, a simple analgesic; gabapentin, primarily used for neuropathic pain; lorazepam, an anxiolytic and sedative; and morphine, a strong opioid analgesic. CLINICAL REASONING: Acute appendicitis can generally be clinically diagnosed based on physical examination and history. The use of analgesics, especially morphine, used to be contraindicated before surgical evaluation based on the belief that it might mask the symptoms, making diagnosis difficult. However, newer studies and guidelines suggest that judicious use of analgesics doesn't interfere with the diagnostic process and can effectively alleviate pain. Assessing the given options, lorazepam, being a sedative, might not be suitable for pain relief, while gabapentin, primarily for neuropathic pain, wouldn't be the ideal choice. Acetaminophen might not be potent enough to alleviate severe appendiceal pain. Morphine would be more effective. CONCLUDING: Since the potential diagnosis is acute appendicitis and patient comfort must be prioritized, using an analgesic to effectively alleviate his pain would be the most appropriate next step. The concern historically associated with the use of analgesics potentially interfering with surgical evaluation is less relevant today since these medications, when used judiciously, can alleviate pain without significantly affecting the diagnostic process. Of the given drug choices, morphine seems to be the best suited to this task. RESPONSE: The choice that best answers the prompt is option (D) Administer morphine, which is the most potent pain reliever of the given options, suitable for the patient's intense abdominal pain.

**AI_A:** (1) RESTATING THE QUESTION: The situation at hand revolves around a 24-year-old man who arrived at the emergency department with right lower quadrant abdominal pain and nausea. His vital signs show he has a fever and an elevated pulse rate. He exhibits tenderness in his right lower quadrant during the physical examination, and wishes to receive pain relief. His medical history is unremarkable, and he doesn't take any medication apart from a multivitamin. However, the surgical consultant can't evaluate him for another two hours. The question essentially asks what the most suitable next step would be in this scenario. (2) PLANNING: To this end, the assumptions that can be made are that the patient has an acute abdominal condition that might necessitate surgical intervention, such as acute appendicitis – as indicated by the location of the pain, elevated temperature and pulse rate. Another assumption is that the patient's pain might affect the physical examination by the surgical consultant in two hours. Hence, any pain relief administered must be decided upon taking into account its potential impact on the evaluation. Acetaminophen, gabapentin, lorazepam, morphine are all analgesics, while the last option suggests informing the patient that analgesics might interfere with the surgical examination. (3) CLINICAL REASONING: If we think about the alternate assumption of which analgesics to give - acetaminophen is a non-opioid analgesic and antipyretic used to treat mild to moderate pain. Gabapentin is an anticonvulsant or antiepileptic drug also used pain relief in neuropathic conditions. Lorazepam, a benzodiazepine, has sedative, anti-anxiety, anticonvulsant, and muscle relaxant properties. Morphine is an opioid used in severe pain conditions. Given the location and nature of the patient's pain, the symptoms align with an acute abdominal situation like appendicitis. It has been shown that administering opioid analgesics to such patients doesn't affect the accuracy of the physical examination or decision-making ability of the physicians during subsequent evaluations. (4) CONCLUDING: Connecting the previously mentioned concerns, the use of morphine could provide effective pain relief to the patient without hampering the surgical evaluation. Its potential to mask physical examination findings has been put under questioning, but recent studies suggest that morphine administration doesn't affect the diagnostic accuracy in cases like acute appendicitis. Therefore, it could be the ideal choice for the maintenance of patient comfort while ensuring proper surgical evaluation. (5) RESPONSE: Hence, the most appropriate next step would be to administer morphine (Option D).

**AI_E:** (1) RESTATING THE QUESTION: The question details a 24-year-old man who came to the emergency department due to sudden right lower quadrant abdominal pain and nausea. He has a slightly high temperature and is in pain. He does not have a significant medical history and no other abnormalities are found on physical examination except for tenderness in the same area as his pain. The surgical consultant is unavailable to assess him for another 2 hours, so the question is asking what should be done next. The options provide different types of medications for consideration, including painkillers like acetaminophen and morphine, a nerve pain medication (gabapentin), an anxiety medication (lorazepam), or explaining to the patient that pain medication might interfere with subsequent surgical evaluation. (2) PLANNING: We would need to think about what the most immediate needs of the patient are, given that there is a significant delay before the surgical evaluation can happen. The first thing to consider is his pain level and if there is a safe way to manage that without interfering diagnosing the cause of his pain. The options suggest both pharmacological and non-pharmacological methods of handling this issue. However, there is a common misunderstanding that opioids, such as morphine, could mask the underlying signs and symptoms and interfere with the diagnosis of acute surgical conditions, thus they might not be a good option. (3) CLINICAL REASONING: The cardinal signs of inflammation that include pain, tenderness, and fever are significant trace indicators that aid in localizing the site of an issue within the abdominal cavity. His localized pain in the right lower quadrant might be due to conditions such as acute appendicitis, a common surgical emergency in young adults. Appendicitis is characterized by an intense, sudden pain, which is also usually accompanied by nausea or vomiting. Moreover, appendicitis typically presents with exquisite pain and tenderness in the right lower quadrant—exactly where the patient is experiencing pain. Although we yet do not have a definitive diagnosis, the possibility of appendicitis looms large. (4) CONCLUDING: Surprisingly, a study published in the "Journal of the American Medical Association" (JAMA) reported pain control in acute abdominal pain doesn't impair the ability to clinically diagnose the disease. The study found that administering analgesia to such patients would not influence the reliability of physical examination or the need to perform unnecessary surgery. As a result, not relieving the patient's pain in a timely manner could actually be detrimental to the patient's well-being. (5) RESPONSE: Therefore, the patient's pain, which is rated 6 on a scale of 10, of course, requires adequate relieving. Considering the possibilities and the fact that the surgical consultant will not be available for the next 2 hours, the most appropriate next step would be to "Administer Morphine" (D), as it will adequately manage the patient's pain without interfering with the diagnostic process in the event of a potential surgical condition like acute appendicitis.

**Facilitator** The consensus statement synthesizes the responses of AI_A, AI_B, AI_C, AI_D, and AI_E as follows: The case presents a 24-year-old man who arrived at the emergency department with sudden right lower quadrant abdominal pain and nausea. His vital signs indicate a fever and an elevated pulse rate, and he exhibits tenderness in his right lower quadrant during the physical examination. The patient's medical history is unremarkable, and he doesn't take any medication apart from a multivitamin. However, the surgical consultant can't evaluate him for another two hours. The question asks what the most suitable next step would be in this scenario. The patient's symptoms suggest an acute abdominal condition, possibly acute appendicitis, given the location and nature of the pain, elevated temperature, and pulse rate. The immediate priority is to manage his pain, which is rated 6 on a 10-point scale. The options provided include various analgesics, such as acetaminophen, gabapentin, lorazepam, and morphine, or explaining to the patient that analgesics might interfere with the surgical examination. Traditionally, the use of analgesics in acute abdominal pain was avoided to maintain the clinical picture. However, recent studies suggest that analgesics, particularly opioids like morphine, do not obscure the diagnosis and can improve patient comfort. They do not reduce the tenderness on palpation, confuse the clinical picture, or delay the diagnosis. Therefore, not relieving the patient's pain in a timely manner could actually be detrimental to the patient's well-being. Given the severity of the patient's pain and the delay before the surgical evaluation, the most appropriate next step would be to administer morphine. This medication will provide the necessary pain relief for the patient while waiting for the surgical consultation. It falls within acceptable practice according to recent evidence and will improve patient comfort without significantly disrupting the ability of the surgical consultant to evaluate the patient's condition.
